# Advanced Cellulose Triacetate-Based Mixed Matrix Membranes Enhanced by Bimetallic Ni-Cu-BTC MOFs for CO_2_/CH_4_ Separation

**DOI:** 10.3390/polym17162258

**Published:** 2025-08-21

**Authors:** Esha Asad, Ayesha Raza, Amna Safdar, Muhammad Nouman Aslam Khan, Humais Roafi

**Affiliations:** School of Chemical and Materials Engineering (SCME), National University of Sciences and Technology (NUST), Islamabad 44000, Pakistan; easad.pse5scme@student.nust.edu.pk (E.A.); mnouman@scme.nust.edu.pk (M.N.A.K.); hroafi.che9scme@student.nust.edu.pk (H.R.)

**Keywords:** mixed matrix membranes, cellulose triacetate, Ni-Cu-BTC, metal–organic frameworks, CO_2_/CH_4_ separation

## Abstract

Cu-BTC (HKUST-1) metal–organic framework (MOF) is widely recognized for its carbon capture capability due to its unsaturated copper sites, high surface area, and well-defined porous structure. This study developed mixed matrix membranes (MMMs) using cellulose triacetate (CTA), incorporating bimetallic Ni-Cu-BTC MOFs for CO_2_/CH_4_ separation, and benchmarked them against membranes fabricated with monometallic Cu-BTC. CTA was selected for its biodegradability, membrane-forming properties, and cost-effectiveness. The optimized membrane with 10 wt.% Ni-Cu-BTC achieved a CO_2_ permeability of 22.9 Barrer at 25 °C and 5 bar—more than twice that of pristine CTA—with a CO_2_/CH_4_ selectivity of 33.8. This improvement stems from a 51.66% increase in fractional free volume, a 49.30% rise in the solubility coefficient, and a 51.94% boost in the diffusivity coefficient. Dual-sorption model analysis further confirmed enhanced solubility and adsorption mechanisms. These findings establish CTA/Ni-Cu-BTC membranes as promising candidates for high-performance CO_2_ separation in natural gas purification and related industrial processes.

## 1. Introduction

The rising energy demands of today’s world have significantly increased natural gas consumption, which currently meets over 22% of global energy requirements [1]. This trend is expected to continue, with global energy consumption projected to rise from 505 Giga BTUs in 2017 to 770 Giga BTUs by 2035 [2]. While natural gas emits less CO_2_ than coal or oil, its growing use, alongside other fossil fuels, contributed to atmospheric CO_2_ levels exceeding 417 ppm by 2022, a 50% rise since pre-industrial times [3]. CO_2_ emissions increased drastically from burning fossil fuels, reaching 36.3 billion tons in 2022 [4]. Methane (CH_4_), a significant component of natural gas, has a high heating value of 55.5 MJ/kg, which makes it an attractive energy source [5]. However, CO_2_ in natural gas reduces its energy density, causes pipeline corrosion, and introduces operational challenges [6]. Therefore, removing CO_2_ is crucial for enhancing purity and improving methane’s energy content and quality.

The conventional technologies used for CO_2_ separation and capture include cryogenic distillation [7], microbial processes [8], absorption [9], and adsorption [10]. Cryogenic distillation is highly energy-intensive and costly [11]; absorption involves high regeneration energy and solvent degradation [12]; adsorption suffers from slow regeneration and moisture sensitivity [13]; and microbial techniques face challenges related to scalability, operational costs, and maintaining microbial stability under varying environmental conditions [14]. In contrast, membrane separation technology has become a widely favored method for this application due to its low energy requirements, cost-effective operation, ability to function continuously, compact design, ease of scale-up, and minimal environmental impact [15]. Among the various types of membranes, polymeric membranes are beneficial over ceramic and inorganic membranes due to their easy fabrication, mechanical robustness, and scalability [16]. These properties ensure their effectiveness in gas separation processes at an industrial scale [17].

One of the most widely used polymers, cellulose acetate (CA), is a thermoplastic material synthesized from cellulose. It can be broadly categorized into two primary forms: cellulose diacetate (CDA) and cellulose triacetate (CTA) [18]. This classification is based on the degree of acetylation, which can vary between 1 and 3, specifying the number of hydroxyl groups that acetyl groups replace on the cellulose repeat unit [19]. Cellulose triacetate (CTA), characterized by the highest degree of acetylation, possesses excellent thermal stability and exhibits a higher affinity for CO_2_ over CH_4_ [20]. It is highly valued for its low cost, non-toxic characteristics, and biodegradable nature, making it a sustainable and safe choice for various applications [21]. It is recognized for its excellent film-forming capacity, in addition to its strong thermal stability and mechanical strength. It offers efficient gas transport characteristics and exceptional chemical and structural stability. CTA membranes remain stable even when exposed to acidic gases such as sulfur dioxide (SO_2_), which makes them well-suited for use in demanding or corrosive environments [22].

In polymeric membranes, enhancing gas permeability typically reduces selectivity, a phenomenon described by Robeson’s upper-bound curve [23,24,25]. To address this trade-off, numerous high-efficiency membranes have been designed and optimized, such as mixed matrix membranes (MMMs) [26], thermally rearranged membranes (TRMs) [27], carbon molecular sieve (CMS) membranes [28], and polymer of intrinsic microporosity (PIM) membranes [29]. Among these, MMMs stand out for merging the ease of polymer processing with the superior performance of inorganic fillers. Incorporating nanoparticles into polymer matrices enhances permeability and selectivity by increasing fractional free volume (FFV) and creating additional pathways for gas molecules [16]. This structural modification increases gas permeability for CO_2_ and H_2_, while also enhancing CH_4_ selectivity [30]. However, weak interactions at the filler–polymer interface may give rise to non-selective voids, negatively affecting membrane performance [31].

Metal–organic frameworks (MOFs) are widely utilized as nanofillers in MMMs, primarily because their tunable pore networks, large specific surface areas, and high porosity enable enhanced gas separation performance [32]. Also, MOFs, with their partially organic structure, are easier to integrate into polymers compared to zeolites [33]. Among MOFs, Cu-BTC (HKUST-1, MOF-199, Basolite C-300) is particularly effective for CO_2_/CH_4_ separation [34]. Cu-BTC consists of divalent copper ions (Cu^2+^) coordinated with benzene-1,3,5-tricarboxylate (BTC) ligands, forming a stable three-dimensional porous framework [35,36,37,38]. Its crystalline structure, with unsaturated copper sites and selective pores, enables strong interactions with CO_2_ molecules, which possess a significant quadrupole moment, enhancing both permeability and selectivity [39]. According to findings by Mu et al. [40], the Cu-BTC MOF was capable of capturing 7.2 millimoles of CO_2_ per gram when tested at 1 bar and 273 K. Under identical testing conditions, MOF-74 showed a lower uptake, reaching only 4.9 millimoles per gram. Cu-BTC is distinguished by its large specific surface area, measured at around 945 m^2^/g [41], a substantial pore volume of roughly 0.70 cm^3^/g [42], and excellent thermal stability, withstanding temperatures up to 350 °C [43]. When Cu-BTC is integrated into polymer matrices, its high surface area and controlled porosity create microporous regions that enhance gas transport and exhibit good CO_2_ affinity. Mahdi et al. [36] reported a CO_2_ permeability of 238.8 Barrer with 15 wt.% NH_2_-Cu BTC in Pebax 1657 and selectivities to 29.72 (CO_2_/CH_4_) and 43.59 (CO_2_/N_2_). A 23% improvement in CO_2_ permeance was achieved by Swati et al. [44] by incorporating 10 wt.% Cu-BTC into polysulfone-based MMMs.

Although single-metal MOFs have received considerable attention for gas separation, research on bimetallic MOFs in this field remains relatively underexplored. Incorporating a secondary metal into the MOF framework—whether during initial synthesis or via post-synthetic modification—can notably influence its porosity, its structural characteristics, and the spatial arrangement of active sites [45,46,47]. These modifications can improve gas adsorption due to synergistic interactions between the two metal centers acting as open sites [48,49,50]. For instance, Zhou et al. [51] showed that incorporating magnesium into MIL-101(Cr) enhanced its capacity to adsorb CO_2_ to 3.28 mmol/g at 1 bar, reflecting a 40% increase over the monometallic MIL-101(Cr). Li et al. [52] reported that a bimetallic MOF comprising copper and zinc (Zn/Cu-BTC) achieved a CO_2_/N_2_ selectivity of up to 17, exceeding that of its monometallic counterparts. By incorporating both titanium and zirconium into the UiO-66 framework, Lau et al. [53] achieved a significant 81% improvement in CO_2_ adsorption performance over the pristine material. Shahbaz et al. [54] integrated bimetallic Zn/Co-ZIF-NH_2_ into 6FDA-DAM polyimide membranes, leading to marked enhancements in the H_2_/CH_4_ and CO_2_/CH_4_ separation performance. Li et al. [55] incorporated rod-like bimetallic Cu_0_._5_Ca_0_._5_-SBMOF into Matrimid^®^ 5218 membranes for CH_4_/N_2_ separation, achieving a selectivity of 17.03 and notable permeability with excellent stability. Khan et al. [56] enhanced composite hollow fiber membranes from polysulfone by incorporating amine-functionalized PEI loaded with copper–magnesium-modified HKUST-1 MOF, achieving a CO_2_/CH_4_ selectivity of 51.

This work investigates the impact of incorporating bimetallic Ni-Cu-BTC MOF into CTA-based MMMs, focusing on the separation performance of CO_2_/CH_4_, and benchmarks their performance against MMMs containing monometallic Cu-BTC. We hypothesize that introducing nickel into the Cu-BTC framework will significantly enhance CO_2_/CH_4_ separation by generating additional active sites, facilitating stronger interactions with CO_2_ molecules, increasing adsorption affinity, and improving structural stability through synergistic effects between the two metal centers. This study is the first to explore CTA/Ni-Cu-BTC MMMs for CO_2_/CH_4_ separation, aiming to improve membrane performance for natural gas purification.

## 2. Experimental Section

### 2.1. Materials

**Figure 1:**
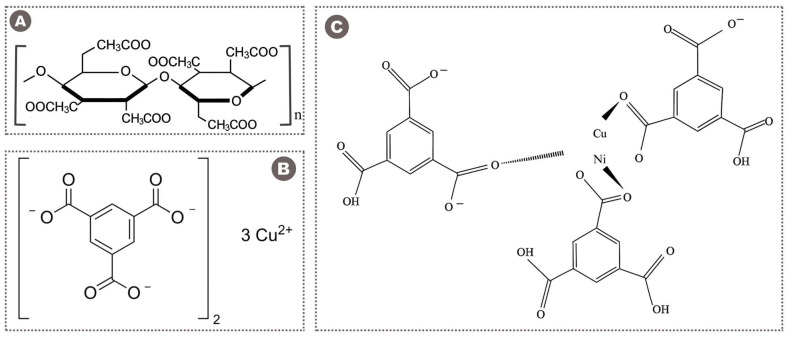
Chemical structures of (**A**) CTA polymer [18], (**B**) Cu-BTC MOF [57], and (**C**) Ni-Cu-BTC MOF [58]. (Adapted with permission from Ref. [58]. Copyright 2025, Elsevier).

For the fabrication of membranes, cellulose triacetate (CTA, DS = 2.84) and the polar aprotic solvent N-methyl pyrrolidone (NMP) were supplied by Sigma-Aldrich (St. Louis, MO, USA). The materials utilized for the MOF synthesis included nickel(II) nitrate hexahydrate, copper(II) nitrate trihydrate, benzene-1,3,5-tricarboxylic acid (BTC), dimethylformamide (DMF), ethanol, and deionized water, all obtained from the same supplier. Each chemical reagent had a purity level of 99.99%, ensuring the reliability of experimental outcomes. For gas permeation testing, high-purity CO_2_ and CH_4_ gases were supplied by Paradise Gases (Islamabad, Pakistan). Figure 1 illustrates the chemical configuration of CTA and the MOFs utilized in this research.

### 2.2. Synthesis Procedures for Cu-BTC and Ni-Cu-BTC MOFs

To synthesize Cu-BTC MOFs, 1.5 g of BTC was dissolved in a solvent mixture comprising equal volumes (22.5 mL each) of DMF and ethanol. In a separate step, 3.11 g of copper(II) nitrate was completely dissolved in 22.5 mL of deionized water under continuous stirring to ensure homogeneity. After obtaining a clear solution of copper nitrate, it was slowly introduced into the solution containing BTC. The synthesized reaction mixture was treated in a Teflon-lined autoclave for 12 h at 120 °C. After cooling, the solid product was collected by filtration and vacuum-dried at 55 °C for 24 h to remove residual solvent. The bimetallic Ni-Cu-BTC MOF was synthesized by introducing equimolar quantities of copper(II) and nickel(II) nitrate hexahydrate into a pre-prepared BTC solution. This mixture was processed using the same temperature and vacuum-drying conditions as applied in the Cu-BTC synthesis procedure to ensure consistency across samples.

### 2.3. Membrane Fabrication

#### 2.3.1. Pristine Cellulose Triacetate (CTA) Membrane

A 12% *w*/*v* solution of CTA in NMP was prepared to fabricate a pristine membrane. Prior to solution preparation, the powdered cellulose triacetate was oven-dried for 5 h at 100 °C to ensure exclusion of any residual moisture content. After drying, the polymer was slowly introduced into NMP and stirred steadily at 45 °C for 24 h for thorough dissolution. Following this, the polymer solution was degassed to eliminate entrapped air bubbles. The homogeneous mixture was then cast evenly onto a cleaned glass slab. To finalize the membrane formation, the cast film was dried overnight in a vacuum oven at 100 °C. After drying, the resulting membrane was carefully detached from the glass using a surgical blade, yielding an intact and defect-free film.

#### 2.3.2. Mixed Matrix Membranes (MMMs)

**Figure 2:**
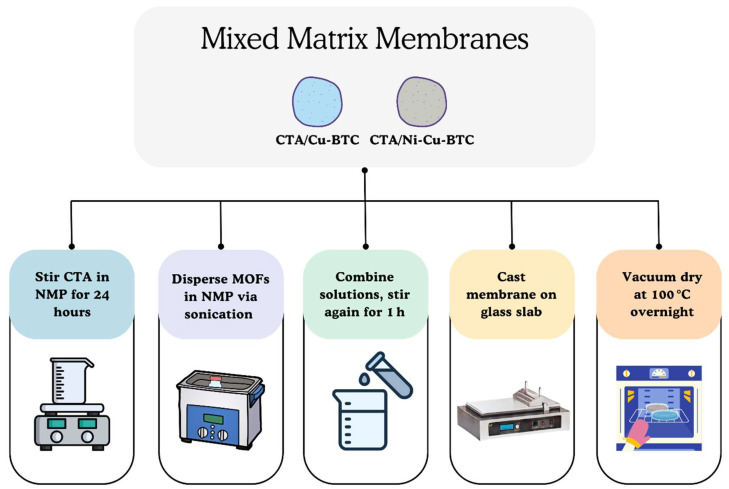
Pictorial representation of MMM fabrication process.

To fabricate Cu-BTC-based MMMs, a homogeneous solution of CTA in NMP was first prepared by stirring continuously overnight. Cu-BTC MOF particles were separately dispersed in NMP and sonicated in a bath sonicator for one hour to ensure uniform nanoparticle distribution. After sonication, the resulting suspension was slowly added to the previously prepared CTA/NMP solution. This blended mixture was then subjected to additional stirring for one more hour to ensure uniform mixing. Then, after uniform casting over a glass plate, the solution underwent vacuum drying at 100 °C for a duration of 12 h. The resulting membranes were then carefully peeled off using a blade. This procedure was followed for all MMM samples, with the stepwise process schematically illustrated in Figure 2. The amount of filler incorporated was determined using a formula:

(1) $$\mathrm{Particle}\;\mathrm{loading}\;(\%)\;=\frac{weight\;of\;the\;particles}{weight\;of\;the\;particles+weight\;of\;the\;polymer}\times 100$$

### 2.4. Membrane Characterizations

To analyze the functional, structural, and morphological features, advanced characterization techniques were used. Fourier Transform Infrared (FTIR) spectra of all fabricated membranes and Ni-Cu-BTC MOF were recorded using a PerkinElmer Spectrum 100 spectrometer (PerkinElmer Inc., Waltham, MA, USA) to investigate functional groups and chemical bonding. The analysis emphasized characteristic vibrational bands, interpreted in comparison with standard spectral references. The spectral analysis was conducted over a wavenumber range spanning from 4000 to 400 cm^−1^. The membranes’ crystalline structure was analyzed with a Bruker D2 Phaser XRD instrument (Bruker AXS GmbH, Karlsruhe, Germany). Scans were performed across a 2θ range of 5° to 35°, allowing the identification of any crystalline phases in the membrane materials. Scanning electron microscopy (SEM) (JSM-6490LA, JEOL Ltd., Tokyo, Japan) was employed to examine the surface and cross-sectional morphology of the membrane samples. For imaging, the samples were fractured under cryogenic conditions using liquid nitrogen and then sputter-coated with a thin platinum layer to improve electrical conductivity. Structural features were examined using SEM at magnifications ranging from 500× to 20,000×. In addition to morphological characterization, an elemental examination of the optimized membrane was undertaken through Energy-Dispersive X-ray Spectroscopy (EDX) using a detector integrated within the microscope. Fractional free volume (FFV) was calculated using Equation (2) to estimate the free volume in each of the fabricated membranes [59,60]:

(2) $$FFV=\;\frac{V-V_{0}}{V}$$

Here, V indicates the molar volume of the cellulose triacetate (CTA) repeating unit, whereas $V_{0}$ is the volume that is occupied by the polymer chains. These values were determined using Equations (3) and (4):

(3) $$V=\frac{M}{\rho }$$

(4) $$V_{0}=1.3\times V_{vdw}$$

The molecular weight M, given in grams per mole (g/mol), corresponds to the repeating unit of CTA. The van der Waals volume ($V_{vdw}$) was calculated based on Bondi’s approach [61]. All volumes—V, $V_{0}$, and $V_{vdw}$—were expressed in cm^3^/mol. The membrane density, ρ, expressed in g/cm^3^, was calculated using Equation (5).

(5) $$\rho =\frac{W_{air}}{W_{air}-W_{liquid}}\times \rho_{0}$$

where $W_{air}$ and $W_{liquid}$ represent the membrane weights in grams, while $\rho_{0}$ indicates the density of ethanol, measured in g/cm^3^. The membranes’ weight was measured both before and after immersion in ethanol for 24 h.

### 2.5. Gas Permeation Measurements

**Figure 3:**
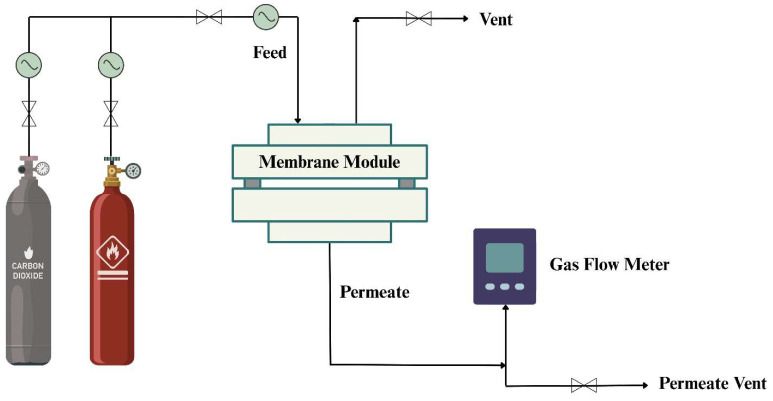
Diagram illustrating the experimental arrangement for gas permeation testing. (Adapted with permission from Ref. [20]. Copyright 2025, Springer Nature).

Membrane performance was assessed through permeation tests using both single- and mixed-gas systems, conducted at 25 °C and a pressure of 5 bar. For both CO_2_ and CH_4_, measurements employed a constant-pressure, variable-volume technique. An equimolar (50:50 *v*/*v*) mixture of CO_2_ and CH_4_ was utilized in the mixed-gas experiments. Each membrane underwent three separate tests to verify consistency, with corresponding standard errors reported. The experimental configuration of the gas permeation setup is shown in Figure 3.

Gas permeability was calculated based on the relationship defined in Equation (6):

(6) $$P_{i}=\frac{Q_{i}\times \Delta l}{\Delta P\times A}$$

where $P_{i}$ denotes the gas permeability expressed in Barrer, where 1 Barrer equals 10^−10^ cm^3^ (STP)·cm/(cm^2^·s^1^·cmHg^1^). $Q_{i}$ represents the volumetric gas flow rate in cm^3^ (STP)/s, Δl is the membrane thickness in centimeters, ΔP signifies the pressure gradient across the membrane in cmHg, and A denotes the effective membrane area, expressed in cm^2^. The CO_2_/CH_4_ selectivity of the membranes was determined using Equation (7):

(7) $$\alpha =\;\frac{P_{{CO}_{2}}\;}{P_{{CH}_{4}}}$$

α denotes the ideal selectivity, while $P_{{CO}_{2}}$ and $P_{{CH}_{4}}$ refer to the respective permeabilities of CO_2_ and CH_4_.

The sorption analysis of CO_2_ for the pristine CTA membrane and the optimized mixed matrix membrane was performed using an H-Sorb 2600 high-pressure adsorption (GOLD APP INSTRUMENT CORP. LTD., HongKong, China) analyzer at 45 °C, with pressures up to 15 bar. Before the sorption studies, the membranes were degassed to eliminate moisture. The solubility coefficient, S (cm^3^(STP)/cm^3^ cmHg), and the diffusivity coefficient, D (cm^2^/s), were calculated at a pressure of 5 bar using Equations (8) and (9), respectively.

(8) $$S=\frac{C}{p}$$

(9) P=S×D 

In these expressions, C  denotes the total amount of gas adsorbed, given in cm^3^ (STP)/cm^3^, and feed pressure is represented by p, measured in cmHg.

The dual-mode sorption model, represented by Equation (10), was employed to further analyze CO_2_ sorption behavior, characterizing gas solubility in glassy polymers via two distinct mechanisms. The initial term in Equation (10) represents gas absorption into the polymer matrix as described by Henry’s law. The second term corresponds to Langmuir adsorption, which explains gas uptake in microvoids or “holes” within the polymer [62,63]. The dual-mode sorption model is mathematically expressed as

(10) $$C=C_{D}+C_{H}=K_{D}p+\frac{{C\prime }_{H}bp}{1+bp}\;$$

Here, C denotes the total gas concentration within the polymer, measured in cm^3^(STP)/cm^3^ polymer. It consists of two parts: $C_{D}$, representing the Henry’s law contribution, and $C_{H}$, arising from Langmuir adsorption—both expressed in the same units. $K_{D}$ is the Henry’s law constant, with units of cm^3^ (STP)/cm^3^ polymer·bar. The term ${C\prime }_{H}$ signifies the Langmuir capacity constant, reflecting the maximum adsorption capacity, while b is the Langmuir affinity constant, indicating adsorption strength, measured in 1/bar. Lastly, p refers to the feed pressure applied during sorption, expressed in bar.

The parameters $K_{D}$, ${C\prime }_{H}$, and b were determined using a graphical method based on the procedure described by Vieth et al. [64]. To evaluate the accuracy, the standard error of the estimate (SEE) was calculated using Equation (11):

(11) $$SEE=\;\sqrt{\frac{\sum_{i}{{(C}_{i,\;exp}-C_{i,\;calc})}^{2}}{n-p}}$$

where $C_{i,\;exp}$ represents the experimental concentration, $C_{i,\;calc}$ denotes the concentration calculated from Equation (10), n represents the total count of data points, and p refers to the number of parameters estimated in the model. SEE was chosen over the correlation coefficient (R^2^) as it provides a more reliable fit measure for nonlinear regression models, including the dual-sorption model [65].

## 3. Results and Discussion

### 3.1. Fourier Transform Infrared (FTIR) Analysis

**Figure 4:**
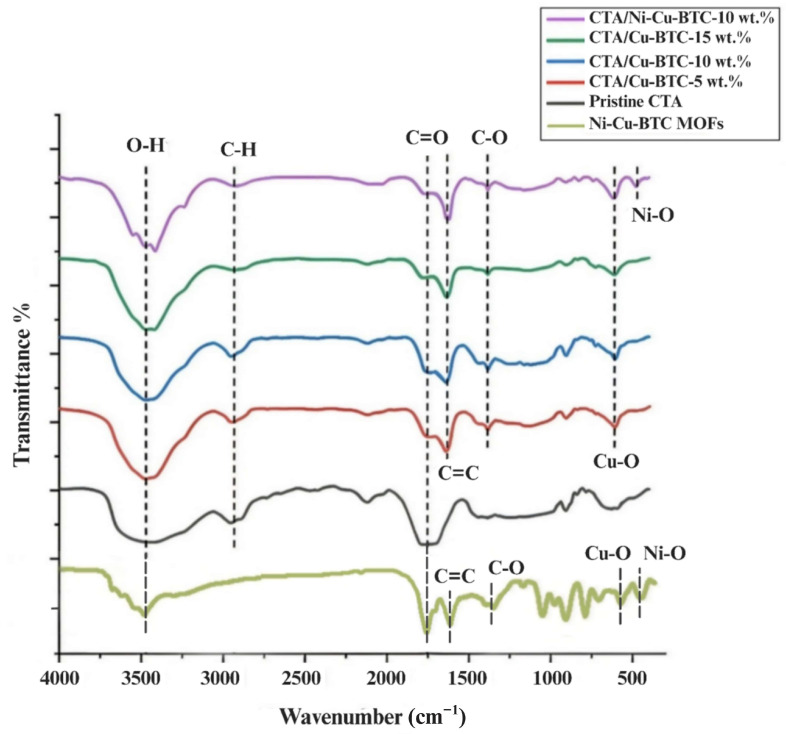
FT-IR spectra of pristine CTA, CTA/Cu-BTC, and CTA/Ni-Cu-BTC membranes, and Ni-Cu-BTC MOFs.

Figure 4 displays the FTIR spectra corresponding to the fabricated membranes and the Ni-Cu-BTC MOFs. All membrane spectra showed distinct bands near 3480 cm^−1^, 2950 cm^−1^, and 1740 cm^−1^, which are indicative of O–H, C–H, and C=O stretching vibrations, respectively [66]. Characteristic peaks at ~1380 cm^−1^ (C–O stretch) and ~610 cm^−1^ (Cu–O stretch) confirm the successful embedding of Cu-BTC MOF into the MMM structure [67]. A sharp peak between 1675 and 1600 cm^−1^ signifies C=C stretching vibrations from benzene rings, indicating the presence of the H_3_BTC linker in the Cu-BTC MOF [68]. The detection of a peak around 470 cm^−1^, corresponding to Ni–O stretching vibrations, confirmed the successful incorporation of nickel into the Ni-Cu-BTC MOF framework [50,69]. Furthermore, the FT-IR spectrum of the Ni-Cu-BTC MOFs shows identical characteristic bands to those reported in the literature [70], validating the structural integrity of the synthesized MOFs. The absence of new peaks suggests that no strong chemical interactions or structural degradation occurred during MOF incorporation, indicating that the MOFs retained their chemical identity within the CTA matrix [71].

### 3.2. X-Ray Diffraction (XRD) Analysis

**Figure 5:**
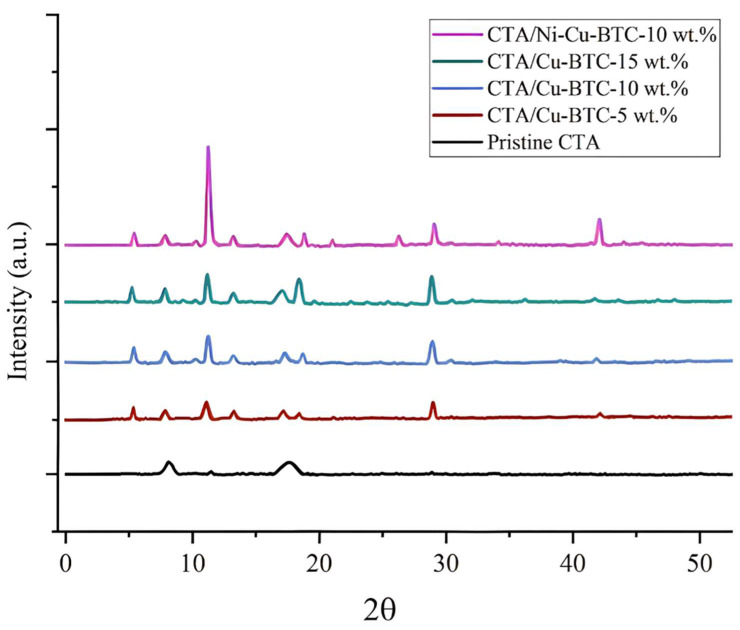
XRD patterns of pristine CTA, CTA/Cu-BTC, and CTA/Ni-Cu-BTC membranes.

Figure 5 presents the XRD patterns, which provide insight into the crystalline phases of the fabricated membrane samples. The pristine CTA membrane exhibited distinct diffraction peaks near 2θ angles of 8° and 18°, indicative of its semi-crystalline nature [20]. The incorporation of Cu-BTC into CTA membranes introduced characteristic peaks around 6.7°, 11.78°, 13.34°, 19.24°, and 28.97°, signifying the presence of the Cu-BTC crystalline phase [72,73]. With increasing Cu-BTC loading, the intensity of these peaks increased, indicating a higher contribution of the crystalline MOF phase within the membrane. Additional peaks around 21.5°, 26.3°, and 43.20° were observed in the CTA/Ni-Cu-BTC-10 wt.% MMM due to Ni incorporation. The prominent peak near 11.78° further confirmed its higher crystallinity, as supported by the literature [41]. This can be attributed to the partial exchange of Ni^2+^ ions into the Cu-BTC structure, which led to an intensified peak. The diffraction patterns of all fabricated MMMs revealed characteristic peaks for both the polymer and the incorporated nanofillers. This indicates that the crystalline phases of both the MOF fillers and the polymer were retained during membrane fabrication.

### 3.3. Scanning Electron Microscopy (SEM) and EDX Analysis

**Figure 6:**
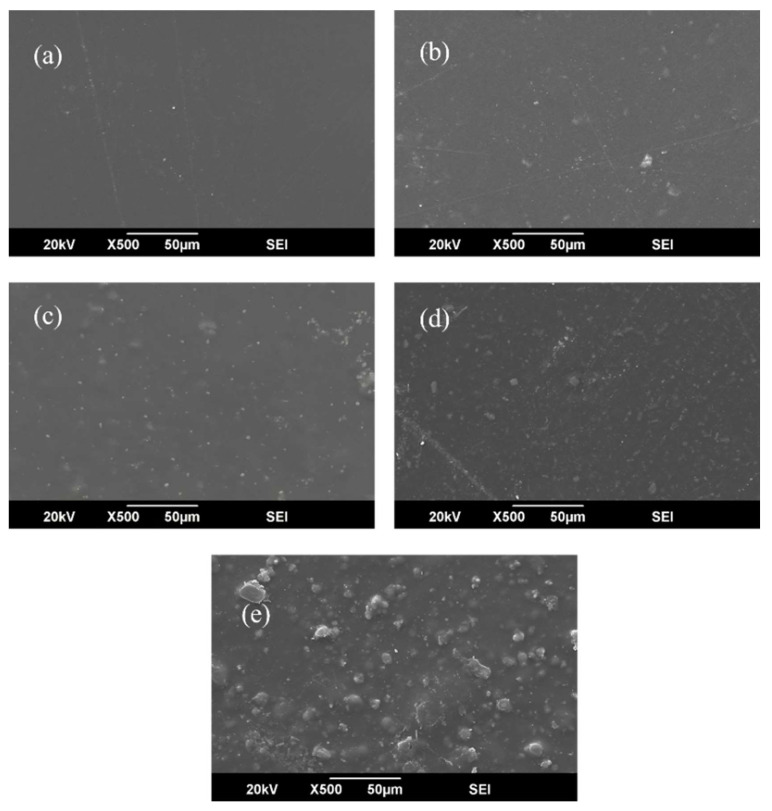
Surface morphology of membranes: (**a**) pristine CTA, (**b**) CTA/Cu-BTC-5 wt.%, (**c**) CTA/Cu-BTC-10 wt.%, (**d**) CTA/Ni-Cu-BTC-10 wt.%, and (**e**) CTA/Cu-BTC-15 wt.%.

**Figure 7:**
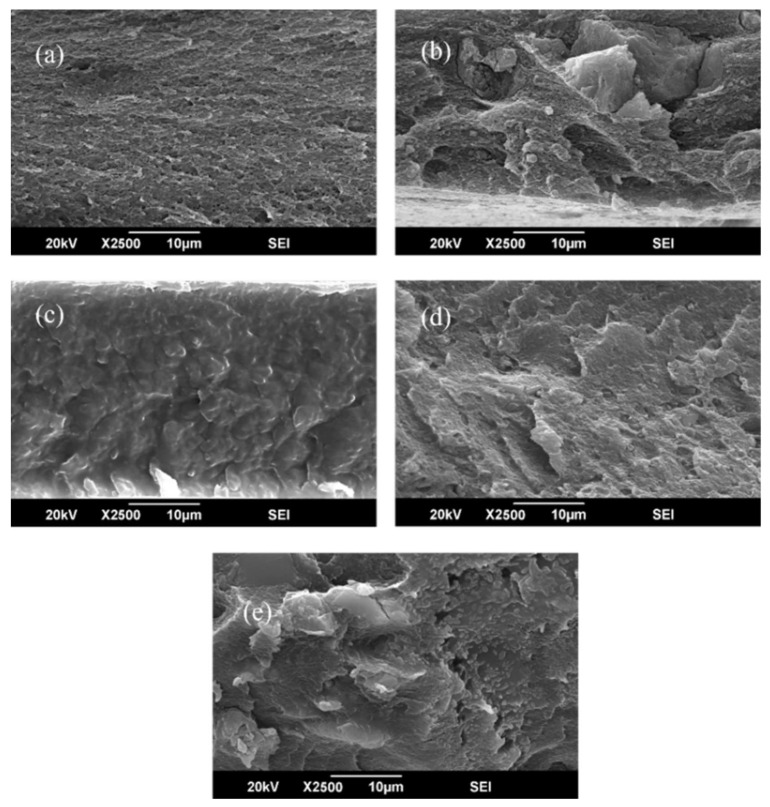
Cross-sectional morphology of membranes: (**a**) pristine CTA, (**b**) CTA/Cu-BTC-5 wt.%, (**c**) CTA/Cu-BTC-10 wt.%, (**d**) CTA/Ni-Cu-BTC-10 wt.%, and (**e**) CTA/Cu-BTC-15 wt.%.

**Figure 8:**
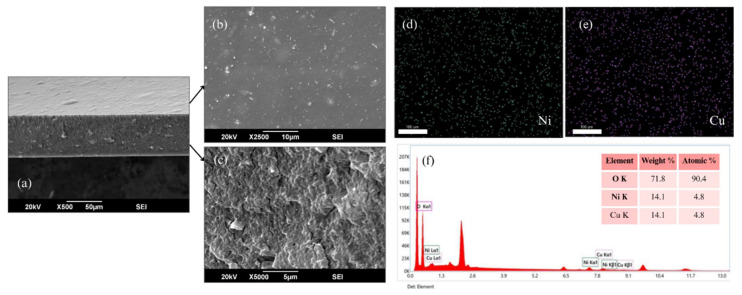
SEM and EDX analysis of the optimized CTA/Ni-Cu-BTC-10 wt.% membrane: (**a**) cross-sectional view at 500×; (**b**) surface morphology at 2500×; (**c**) cross-sectional view at 5000×; (**d**) EDX mapping of Ni distribution (green); (**e**) EDX mapping of Cu distribution (purple); and (**f**) EDX spectrum with corresponding elemental composition (weight % and atomic %).

The cross-sectional and surface microstructures of all fabricated membranes at different filler concentrations are presented in Figure 6 and Figure 7. The detailed morphology of the optimized CTA/Ni-Cu-BTC-10 wt.% membrane, along with the corresponding EDX elemental mapping, is shown in Figure 8. All the fabricated membranes exhibited asymmetry, with loosely packed structures between the dense and defect-free skin layer. Moreover, the incorporated nanoparticles were well-dispersed, showing no signs of agglomeration up to 10 wt.% loading. This observation was further confirmed by EDX analysis of the optimized membrane. Elemental mapping revealed that Ni (green) and Cu (purple) from the bimetallic MOF were uniformly distributed across the CTA matrix surface. There was no evidence of agglomeration, indicating homogeneous nanoparticle dispersion even at 10 wt.% loading. These results are primarily due to the favorable interfacial interactions and high compatibility between Cu-BTC and the CTA polymer matrix. The EDX spectra further confirmed a 1:1 atomic ratio of Ni to Cu, verifying the intended bimetallic composition in the membrane. However, when the filler concentration was raised from 10 to 15 wt.%, signs of agglomeration appeared, likely caused by the polymer’s limited capacity to uniformly distribute the excess filler, leading to particle clustering [74]. According to Zhang et al. [75], disparities in physical characteristics between the polymer and MOF crystals may lead to particle agglomeration. Such agglomeration frequently results in the formation of non-selective voids within the membrane and may also contribute to increased brittleness. These findings highlight the need to carefully optimize MOF loading to balance improved separation efficiency with mechanical stability [76].

### 3.4. Fractional Free Volume (FFV) Analysis

Table 1 provides a summary of the fractional free volume (FFV) and density measurements for all fabricated membranes. The values of the pristine CTA membrane aligned well with previously reported data [77]. Integrating Cu-BTC and Ni-Cu-BTC MOFs into the CTA matrix notably increased the membranes’ fractional free volume (FFV). A notable 82.78% rise in FFV was observed when Cu-BTC loading increased from 0 to 15 wt.%. This enhancement is mainly due to the disruption of the regular polymer chain arrangement, leading to the creation of extra free volume within the membrane matrix [78]. Overall, greater free volume typically results in lower density, as evidenced by a 14.75% decline—from 1.186 g/cm^3^ to 1.011 g/cm^3^—when the Cu-BTC content was increased from 0 to 15 wt.%. A similar trend was observed for Cu-BTC/Matrimid MMMs [79]. These findings suggest that while moderate increases in FFV improve gas separation, excessive FFV, as seen at higher MOF loadings, may reduce separation performance due to the formation of non-selective gas transport pathways.

### 3.5. Gas Permeation Analysis

**Figure 9:**
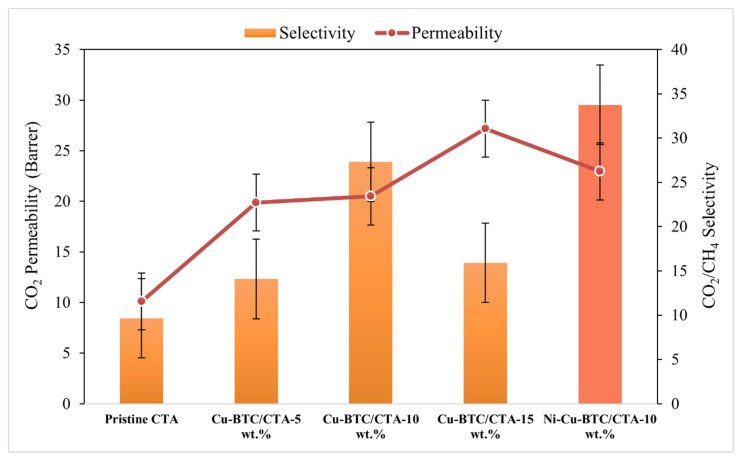
CO_2_/CH_4_ separation efficiency of CTA-based membranes with varying filler content.

**Figure 10:**
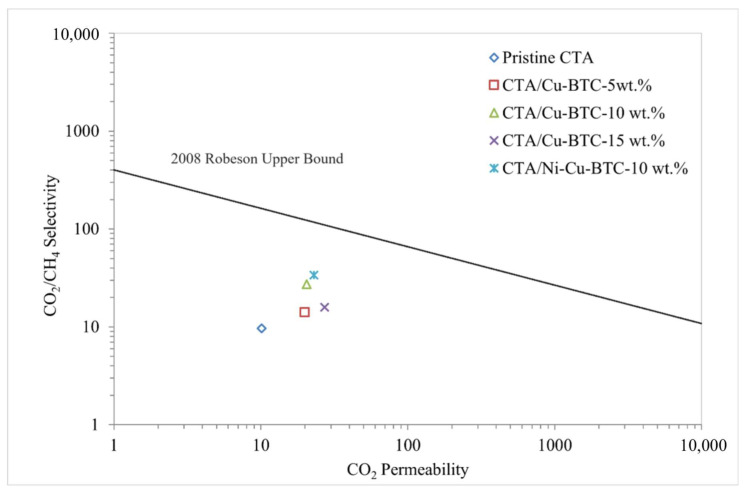
Gas separation performance of pristine CTA and fabricated MMMs against the 2008 Robeson upper bound.

Figure 9 depicts the impact of varying Cu-BTC MOF concentrations on the gas separation efficiency of the CTA membrane. The incorporation of Cu-BTC into the CTA framework notably boosted CO_2_ permeability. However, an up-and-down trend was observed in CO_2_/CH_4_ selectivity. Generally, introducing fillers into a polymer matrix disrupts the polymer chain alignment, which increases the fractional free volume (FFV) and creates additional non-selective diffusion channels for gas molecules. Despite this, increasing the Cu-BTC content from 0 to 10 wt.% significantly boosted CO_2_ permeability by 102.97%. Moreover, CO_2_/CH_4_ selectivity improved by 184.37%, a trend attributed to Cu-BTC’s high affinity for CO_2_ molecules. The increase in permeability with greater filler loading is further supported by FFV analysis (Table 1), which indicates disrupted polymer chain alignment. The observed selectivity improvement may be attributed to the superior molecular sieving capability and crystalline structure of Cu-BTC MOF, characterized by unsaturated copper sites and well-defined pores that favor the adsorption of CO_2_ molecules [39]. However, the decline in selectivity at 15 wt.% can be attributed to (1) particle agglomeration at higher loadings [80], as observed in SEM analysis, (2) excessive fractional free volume (FFV) [81], and (3) potential mechanical instability or polymer rigidification, causing microstructural defects in the membrane [82]. Based on these findings, a bimetallic Ni-Cu-BTC was introduced at an optimized loading of 10 wt.% in the CTA matrix to further investigate gas separation performance. The findings showed a 11.71% boost in CO_2_ permeability, increasing from 20.5 to 22.9 Barrer, and a 23.81% improvement in CO_2_/CH_4_ selectivity, which rose from 27.3 to 33.8. Figure 10 contrasts the performance of pristine CTA and MMMs with Cu-BTC and Ni-Cu-BTC MOFs against the 2008 Robeson upper bound. This benchmark highlights the balance between gases.

**Figure 11:**
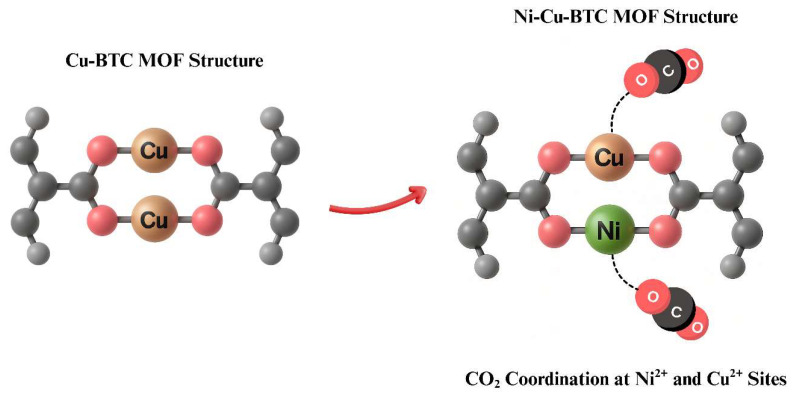
Structural transformation from Cu-BTC MOF to the bimetallic Ni-Cu-BTC MOF, along with CO_2_ adsorption behavior.

The observed improvement in gas separation efficiency is likely due to the partial substitution of Cu^2+^ with Ni^2+^ ions, which strengthens interactions at the unsaturated metal sites. As a result, the membrane exhibited enhanced CO_2_ adsorption capacity and improved molecular affinity [41,46]. Moreover, the addition of nickel promoted a more uniform distribution of active sites, improving the molecular sieving properties and optimizing the pore structure [46,83]. As a result, CO_2_ diffusion was enhanced while CH_4_ diffusion was restricted, leading to superior gas separation performance. Figure 11 illustrates the structural modification from Cu-BTC MOF to bimetallic Ni-Cu-BTC MOF, as well as CO_2_ adsorption at both Cu and Ni sites [84]. This substitution yielded a bimetallic MOF with Ni^2+^ and Cu^2+^ active sites, which enhanced CO_2_ coordination and improved the membrane’s adsorption capability [41,46,83].

In Table 2, the separation performance of pristine CTA and CTA/Ni-Cu-BTC-10 wt.% membranes is compared under mixed-gas conditions using an equimolar CO_2_/CH_4_ (50:50) feed, highlighting the effect of MOF incorporation on gas selectivity and permeability. Under these experimental conditions, CO_2_ permeability shows a marked reduction compared to single-gas measurements, whereas CO_2_/CH_4_ selectivity increases. The reduction in permeability is primarily linked to the competitive sorption and diffusion interactions between CO_2_ and CH_4_, which interfere with the transport process when both gases coexist [85,86]. These observations align with previously published studies [87,88,89,90,91,92]. The higher condensability and stronger interaction of CO_2_ with the CTA matrix enable CO_2_ to preferentially occupy sorption sites and dominate diffusion pathways, thereby restricting CH_4_ movement and enhancing the overall CO_2_/CH_4_ selectivity [93].

### 3.6. Sorption Analysis

**Figure 12:**
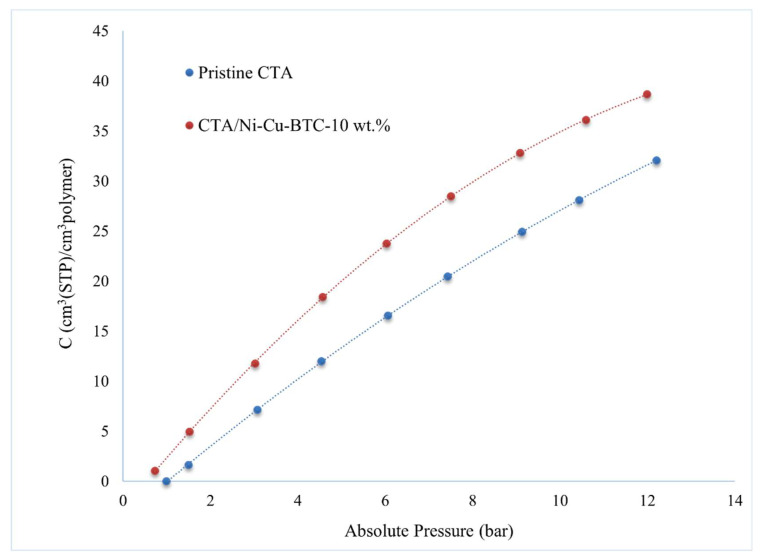
CO_2_ sorption isotherms for the pristine CTA and CTA/Ni-Cu-BTC-10 wt.% membranes measured across varying feed pressures.

Figure 12 shows the CO_2_ sorption behavior for pristine CTA and CTA/Ni-Cu-BTC-10 wt.% membranes, highlighting the dual-mode interaction mechanisms described by the Langmuir–Henry model. Table 3 summarizes the key findings. The optimized membrane incorporating 10 wt.% Ni-Cu-BTC showed a 49.30% increase in the solubility coefficient and a 51.94% enhancement in the diffusion coefficient relative to the pristine CTA membrane. The values for the pristine CTA membrane align closely with the previously reported literature, with slight differences attributed to variations in temperature and acetylation degree [94]. The integration of Ni-Cu-BTC MOF significantly improved the Langmuir capacity constant (${C\prime }_{H}$) by 15.04%, Henry’s coefficient ($K_{D}$) by 14.81%, and the hole affinity constant (b) by 65%. The increase in ${C\prime }_{H}$ was primarily due to the disruption of polymer chain packing caused by the incorporation of Ni-Cu-BTC particles, which created extra free volume and consequently improved CO_2_ sorption capacity. According to Tsujita et al. [95] the presence of unrelaxed volumes or microvoids—both influenced by pressure and temperature—significantly impacts the Langmuir capacity constant (${C\prime }_{H}$). $K_{D}$ and b increased due to improved polymer–gas interactions, reflecting enhanced CO_2_ solubility and sorption affinity in the membrane [96]. Enhanced separation efficiency resulted mainly from the strong affinity between CO_2_ molecules and the active sites generated by the Ni-Cu-BTC MOF, particularly the coordinatively unsaturated Ni^2+^ and Cu^2+^ metal sites, which enhanced the membrane’s affinity for CO_2_ and resulted in higher uptake [41]. Furthermore, the polar characteristics of the CTA/Ni-Cu-BTC membrane contribute notably to its increased affinity for CO_2_. The acetate and ester groups in CTA possess dipolar characteristics, which interacted with the quadrupolar CO_2_ molecules. These dipole–quadrupole interactions increased the solubility of CO_2_ within the membrane matrix [97]. The incorporation of Ni-Cu-BTC MOFs significantly enhanced the membrane’s affinity for CO_2_ molecules by introducing additional polar functional groups and metal coordination sites. Such features facilitated stronger affinity between the membrane matrix and CO_2_ molecules, promoting selective gas sorption and transport. The rise in the diffusion coefficient is likely due to the MOF’s inherent porosity and large surface area, which facilitated multiple pathways for gas movement and increased the membrane’s available free volume. Furthermore, incorporating MOF particles disturbed the regular arrangement of polymer chains, which reduced resistance to gas transport and promoted more rapid CO_2_ diffusion through the membrane [78].

### 3.7. Benchmark with the Literature

Table 4 presents a performance comparison of the newly developed CTA/Ni-Cu-BTC mixed matrix membranes with previously reported CTA-based MMMs and composite materials for single-gas separation. The CTA/Ni-Cu-BTC-10 wt.% membrane demonstrated enhanced CO_2_ permeability and higher CO_2_/CH_4_ selectivity compared to several previously reported membranes. This improvement is primarily due to the incorporation of bimetallic MOFs, which enhanced gas transport by creating better pathways for CO_2_ diffusion while retaining molecular sieving properties.

## 4. Conclusions

In this study, MMMs comprising CTA/Cu-BTC and CTA/Ni-Cu-BTC were prepared via the solution casting technique. Their gas separation performance was assessed across different filler loadings. The findings resulted in the following key conclusions:

(a) The increase in CO_2_ permeability observed at a filler concentration of 10 wt.% loading can be primarily attributed to two key factors: (1) the strong interactions between quadrupolar CO_2_ molecules and the Lewis acidic Cu^2+^ centers present in the Cu-BTC structure, and (2) the enhancement in fractional free volume resulting from the disruption of the polymer matrix structure.
(b) However, CO_2_/CH_4_ selectivity exhibited a fluctuating trend. This trend could result from particle agglomeration at higher loadings and excessive fractional free volume (FFV), which may create non-selective pathways. Therefore, optimized loading is required to fabricate higher-performance CTA/Cu-BTC MMMs.
(c) Pure gas permeation analysis revealed that the optimized bimetallic CTA/Ni-Cu-BTC mixed matrix membrane containing 10 wt.% filler showed a 126.7% increase in CO_2_ permeability along with a 252.1% enhancement in CO_2_/CH_4_ selectivity relative to the pristine CTA membrane. Further validation through mixed-gas separation using a 50:50 CO_2_/CH_4_ mixture confirmed these enhancements, with CO_2_ permeability rising by 91.7% and selectivity improving by 154.8%. This improved performance is primarily due to the synergistic effects introduced by the Ni-Cu-BTC framework, which contributes additional unsaturated metal sites, a higher surface area, and a well-structured pore network—factors that collectively enhance CO_2_ interaction and diffusion.
(d) The enhancement in gas separation performance of CTA/Ni-Cu-BTC-10 wt.% MMM is primarily the result of a 49.30% increase in the solubility coefficient and a 51.94% rise in the diffusion coefficient compared with the pristine membrane. This improvement likely arises from the excellent molecular sieving capability and high surface area of the dual-metal framework. Additionally, the inclusion of Ni-Cu-BTC MOF improved the Langmuir capacity constant (${C\prime }_{H}$) by 15.04%, Henry’s coefficient ($K_{D}$) by 14.81%, and the hole affinity constant (b) by 65%, highlighting significant enhancements in gas sorption and interaction properties.
(e) The FFV analysis supports the permeation and sorption trends, with CTA/Ni-Cu-BTC-10 wt.% showing a notable increase in FFV (0.229) relative to the pristine CTA membrane (0.151).

Therefore, the fabricated CTA/Ni-Cu-BTC MMM demonstrates considerable potential to enhance gas separation efficiency for CO_2_/CH_4_ separation.

## Figures and Tables

**Table 1:** FFV and density measurements of pristine CTA, CTA/Cu-BTC, and CTA/Ni-Cu-BTC membranes.

Membranes	Density (g/cm^3^)	FFV
Pristine CTA	1.186 ± 0.10	0.151 ± 0.12
CTA/Cu-BTC-5 wt.%	1.143 ± 0.13	0.181 ± 0.13
CTA/Cu-BTC-10 wt.%	1.096 ± 0.11	0.215 ± 0.10
CTA/Cu-BTC-15 wt.%	1.011 ± 0.12	0.276 ± 0.12
CTA/Ni-Cu-BTC-10 wt.%	1.076 ± 0.14	0.229 ± 0.13

**Table 2:** Comparison of CO_2_/CH_4_ mixed-gas separation efficiency between pristine CTA and CTA/Ni-Cu-BTC-10 wt.% membranes.

Membranes	CO_2_ Permeability (Barrer)	CO_2_/CH_4_ Selectivity
Pristine CTA	9.6 ± 0.11	15.7
CTA/Ni-Cu-BTC-10 wt.%	18.4 ± 0.12	40.0

**Table 3:** Calculated pure gas CO_2_ permeabilities, solubility coefficients, diffusivity coefficients, $K_{D},{C\prime }_{H},b$, and the standard error of estimate for the pristine CTA membrane and the optimized CTA/Ni-Cu-BTC-10 wt.% mixed matrix membranes.

Membranes	CO_2_ Permeability (Barrer)	Solubility Coefficient (10^−2^ cm^3^(STP)/cm^3^ cmHg)	Diffusivity Coefficient (10^−8^ cm^2^/s)	$K_{D}$	${C\prime }_{H}$	b	Standard Error of Estimate
Pristine CTA	10.1 ± 0.13	3.57	2.83	1.62	16.02	0.20	1.816
CTA/Ni-Cu-BTC-10 wt.%	22.9 ± 0.14	5.33	4.30	1.86	18.43	0.33	1.808

**Table 4:** Benchmarking CO_2_/CH_4_ separation performance of CTA/Ni-Cu-BTC-10 wt.% MMM against reported membranes.

Membranes	Pressure (Bar)	Temp. (°C)	CO_2_ Permeability (Barrer)	CO_2_/CH_4_ Selectivity	Ref.
CDA-CTA	11	35	14	32	[98]
CTA-GO	1.5	25	11.29	34.22	[23]
CTA/PSf	4	25	11.20	30.70	[20]
CTA/[emim][BF_4_]	4	35	12	20	[99]
CTA-CeO2-0.32 wt.%	1 (torr)	25	6.89	24.61	[100]
ZIF-90-(1.5)-CTA	7	25	46.5	10.1	[101]
ZIF-8/CA-10 wt.%	2	25	9.5	15.3	[32]
CA-γ-CD-MOF-0.8 wt.%	5	25	13	35.5	[102]
PBS-CTA-10 wt.%	1.5	25	3.5	35	[103]
PSF-PES-Cu-BTC (52%-28%-20%)	3	25	3.90	20.52	[104]
CTA/Ni-Cu-BTC-10 wt.%	5	25	22.9	33.8	This work

## Data Availability

Supporting data for this study can be obtained from the corresponding author * upon reasonable request.
